# The Inference of the Evolution of Immune Traits as Constrained by Phylogeny: Insight into the Immune System of the Basal Diapsid

**DOI:** 10.3390/ani12182482

**Published:** 2022-09-19

**Authors:** Jorge E. López-Pérez, Brian I. Crother, Christopher M. Murray

**Affiliations:** Department of Biological Sciences, Southeastern Louisiana University, Hammond, LA 70402, USA

**Keywords:** toll-like receptors, diapsida, reptilia, immune, evolution

## Abstract

**Simple Summary:**

In light of emerging pathogenic threats affecting wildlife, it is important to broaden the current understanding of immune system function, development, and evolution. The relation of descent of immune traits is critical to understand the ability of organisms to handle pathogens. Here, we explore the evolution of toll-like receptors (TLRs), a series of receptors crucial to the initial immune response in reptiles. Our analysis revealed that the common ancestor may have had an immune system that lacked two receptors: TLR 15, a receptor uniquely present in Reptilia, and TLR 13, a receptor important in the recognition of pathogens. Additionally, our analysis showed a dynamic evolution for various TLRs, likely attributed to redundancies in function.

**Abstract:**

Among vertebrates, some of the most vulnerable taxa to emergent fungal pathogens are members of Reptilia. In light of the growing threat of emergent fungal pathogens affecting wildlife, it is important to broaden the current understanding of immune system function, development, and evolution. The homologous condition of a trait is necessary in order to study its evolution, as such, homology is necessary in the study of immunological evolution. Here, we explore the evolution of toll-like receptors (TLRs), a series of homologous receptors crucial to the initial immune response. The homologous condition of TLR genes provides a unique system in which to explore the evolution of the TLR; using a Reptilian phylogeny, we elucidate the immune condition of the basal diapsid. Our analysis revealed that the basal diapsid may have had an immune system that lacked two receptors: TLR 15, a receptor uniquely present in Reptilia, and TLR 13, a receptor important in the recognition of nucleic acid motifs. Additionally, our analysis showed multiple losses and convergences for various TLRs, likely attributed to redundancies in receptor function. Further exploration into the immune condition of extinct taxa may shed light on the evolution of the reptilian immune system.

## 1. Introduction

The contemporary explosion of infectious diseases affecting wildlife is necessitating biologists to further understand immune function in non-model organisms. Notable infectious diseases such as Chytridiomycosis, Snake-fungal disease, Upper-respiratory tract infections, and White-nose syndrome, among many others, are causing damaging losses to wildlife populations across the globe. The increased convenience of contemporary travel has facilitated the global spread of diseases [1]. Currently, herpetofaunal taxa are among some of the most threatened taxa, as amphibians and testudines fall high on the list of most threatened taxonomic groups [2,3]. Already suffering from increasing anthropomorphic pressures such as habitat destruction, habitat encroachment, and the pet trade, these groups are simultaneously succumbing to the threat of infectious diseases as well. As such, it is becoming increasingly more important to understand the function of the immune system and its ability to respond to foreign pathogens. Alongside efforts to understand immune function, significant efforts have also been made to peer Into the evolution of the immune system [4]; therefore, it is important to explore the homology of immune traits across taxa in order to gain a glimpse into the evolution and maintenance of immunological traits.

Immune function can be generally separated into two different primary branches: the innate and acquired systems. The acquired immune system is responsible for developing a diverse arsenal of immunoglobulins and recognizing their respective pathogens; conversely, the innate immune system provides rapid responses to foreign bodies without the need for prior exposure [5]. Recognition of foreign bodies or damage to the self—known as pathogen-associated molecular patterns (PAMPs) or damage-associated molecular patterns (DAMPs), respectively—can be identified by the immune system through pattern-recognition receptors (PRRs). There are various types of PRRs; however, for the purpose of this study we focus on Toll-like receptors (TLR). Toll-like receptors are transmembrane proteins characterized by an extracellular leucine-rich repeat (LRR) domain, which allows for ligand recognition- and an intracellular Toll/interleukin-1 receptor (TIR) domain [6,7]. Toll-like receptors are typically expressed on immune cells such as macrophages and neutrophils [7], and function through downstream signaling pathways. The signal pathway begins with the recognition of a ligand by the LRR domain, leading to a cascade of interactions between adaptor proteins, protein kinases, and transcription factors, resulting in the activation of genes which regulate the major histocompatibility complex (MHC), cytokines, and chemokines [7].

Prior studies on the evolution of TLRs have found that the presence of TLR genes is highly conserved throughout animal life. Genes for both Toll and Toll-like receptors have ancient evolutionary origins dating back to the appearance of eumetazoans [6,8]. However, the appearance of TLRs did not occur until the evolution of deuterostomes; protostomes and cnidarians carry genes for the ancestral form of TLR, the Toll protein—a ligand activated protein that initiates the production of antimicrobial peptides, first discovered in *Drosophila melanogaster* [8,9]. Within Deuterostomia, the diversity of TLR genes varies greatly, with chordates having a much lower number of genes (64) relative to echinoderms (253) [8]. With the development of acquired immunity, the number of TLR genes greatly decreases; with vertebrates having between 10–20 TLR genes [8], vertebrate TLR genes are thought to have quickly diversified [10]. Based on the homology of TLR genes, Roach et al. [11] (further expanded upon by [10]) found that vertebrate TLR genes can be grouped into six major families, each recognizing a unique PAMP. For example, the TLR 4 family specifically recognizes lipopolysaccharides (LPS), an important component and exotoxin found on outer-membrane of gram-negative bacteria. It is thought that TLR function is due to strong selective pressures for maintenance of function rather than mutations, as the phylogeny of TLRs recapitulates the phylogeny of species [11]. The conservation or loss of TLR genes has been explored throughout a variety of taxa; however, it is typically done at the class level [10,11]. 

Due to the homology present in TLR genes, inferences into the evolutionary history of these receptors can be made. In this study we aim to explore TLR evolution within Reptilia, as it has not been previously done. While other studies have included Reptilia at a coarse scale among vertebrates, TLR evolution within this clade is critical in the context of emerging pathogens. Here, we hypothesize large variation of TLR genes among lineages, as they inhabit diverse environments, characterized by losses and subsequent maintenance. Further exploration into evolution of TLRs in reptiles may aid in understanding the current immune traits expressed in reptiles. Using character optimization of TLR genes present in Reptilia, we can infer what the basal diapsid immune function might have been.

## 2. Materials and Methods

Using available literature, the presence and/or absence of TLR genes were collected for all TLR genes currently recognized in non-avian members of Reptilia from supplementary data in [10]. Using character optimization techniques, the presence of TLR genes was optimized onto a phylogeny including members of Mammalia as the outgroup clade and both avian and non-avian reptiles (*Equus caballus*, *Mus musculus*, *Homo sapiens*, *Sphenodon punctatus*, *Gekko japonicus*, *Pogona vitticeps*, *Anolis carolinensis*, *Ophiophagus hannah*, *Thamnophis sirtalis*, *Protobothrops mucrosquamatus*, *Python bivittatus*, *Gopherus agassizii*, *Chrysemys picta*, *Chelonia mydas*, *Pelodiscus sinensis*, *Gavialis gangeticus*, *Alligator sinensis*, *Rhea americana*, *Gallus gallus*, *Anas platyrhynchos*). Our phylogeny was created using various hypotheses on vertebrate evolution [12,13,14,15]. All analyses, including the creation of a matrix, were performed in Mesquite (v. 3.61) [16] using a most parsimonious reconstruction framework. The traits were optimized using accelerated (ACCTRAN), delayed (DELTRAN), and Mixed optimization procedures for each trait, which may generate different outcomes depending on the algorithm [17,18]. 

## 3. Results

**Figure 1:**
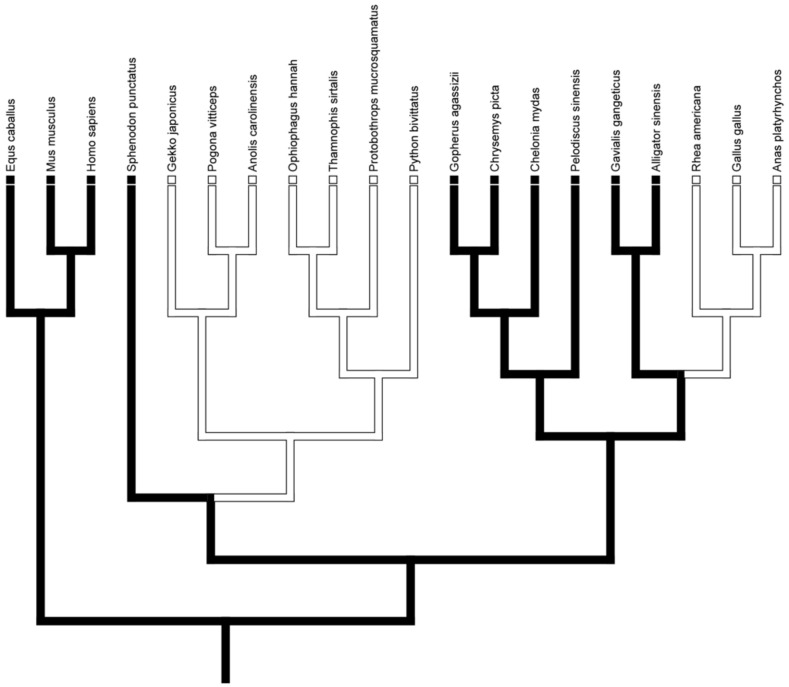
The character optimization of TLR 8. All optimization algorithms yielded an unequivocal inference for TLR 8 genes; the basal diapsid possessed TLR 8, but it was subsequently lost in Lepidosauria and Aves.

The optimization of TLR genes onto a general phylogeny of Reptilia presented various hypotheses for the independent conservation and loss of these genes. Genes for TLR 1–5 and 7 were present in all taxa unequivocally throughout ACCTRAN, DELTRAN, and mixed trees, suggesting the presence of this TLR suite in the basal diapsid and subsequent conservation in all descendants. Interestingly, the basal diapsid possessed TLR 8 but it was subsequently lost in Lepidosauria and Aves and all transformations were unequivocal (Figure 1).

**Figure 2:**
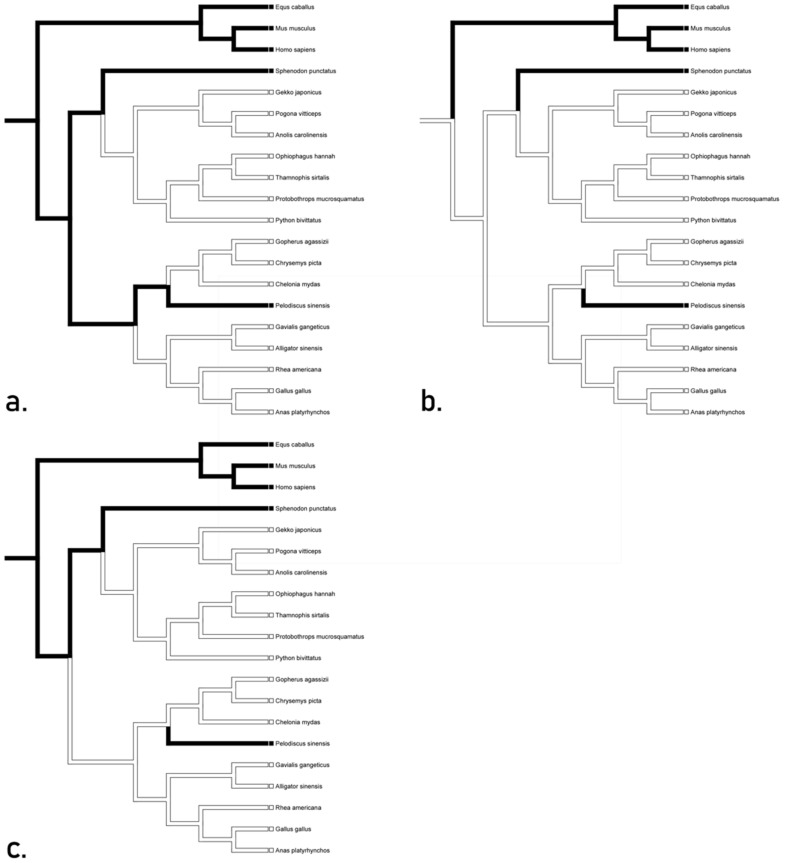
Character optimization of TLR 9 using accelerated delayed, and mixed transformations unequivocally suggesting a loss in squamates, testudines, and crocodilians. The accelerated (**a**) transformation suggests conservation in *Sphenodon* and *Pelodiscus*, while the delayed (**b**) transformation suggests convergence between Mammalia and the aforementioned taxa. The mixed transformation (**c**) suggests conservation in *Sphenodon* and convergence in *Pelodiscus*.

All transformations of TLR 9 unequivocally suggested a loss in squamates, testudines, and crocodilians with outliers being *Sphenodon* and *Pelodiscus* (Figure 2). The accelerated transformation suggests conservation in *Sphenodon* and *Pelodiscus*, while the delayed transformation suggests convergence between Mammalia and the aforementioned taxa. The mixed transformation suggests conservation in *Sphenodon* and convergence in *Pelodiscus*. These hypotheses suggest the presence of TLR 9 gene in the basal diapsid. We recognize the accelerated tree (Figure 2a) as the most likely hypotheses, as it is easier to lose these genes rather than to gain them. 

**Figure 3:**
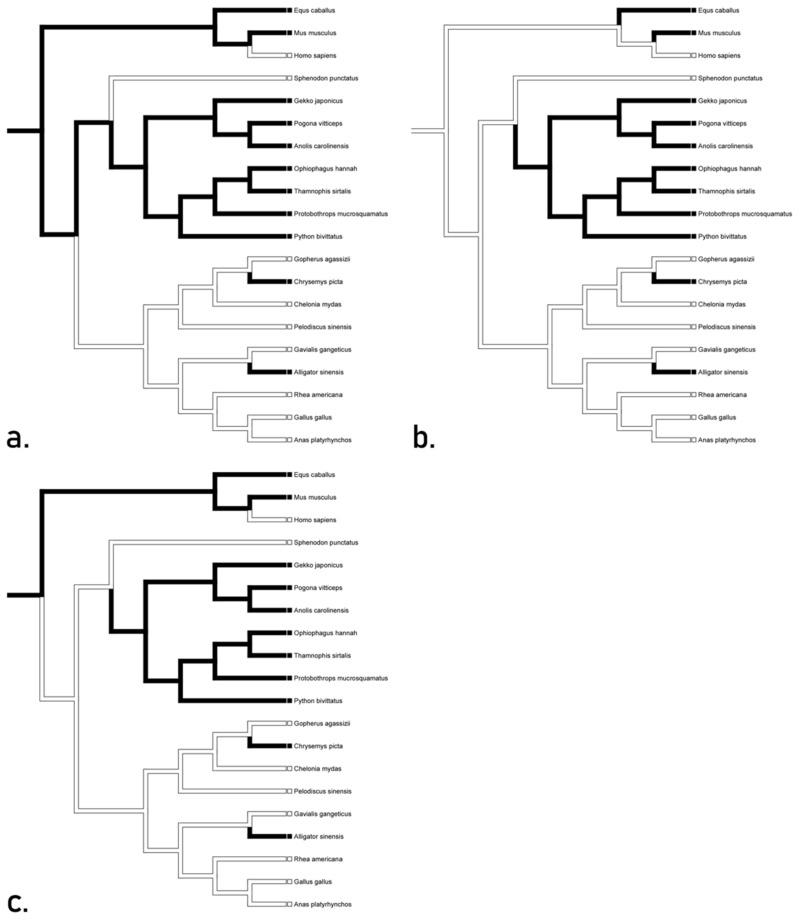
Character optimization of TLR 13 using accelerated (**a**) delayed (**b**), and mixed (**c**) transformations supporting convergence between *Chrysemys* and *Alligator* for the gene in all three proposed trees, as well as a loss in *Sphenodon*. Accelerated transformation suggests conservation in squamates and convergence between *Chrysemys* and *Alligator*, while delayed and mixed transformations suggest a reversal for the gene in squamates.

Analyses for TLR 13 supports convergence between *Chrysemys* and *Alligator* for the gene in all three proposed trees, as well as a loss in *Sphenodon* (Figure 3). Accelerated transformation suggests conservation in squamates and convergence between *Chrysemys* and *Alligator*, while delayed and mixed transformations suggest a reversal for the gene in squamates. These hypotheses indicate that the gene for TLR 13 may not have been present in the basal diapsid. We recognize the ACCTRAN tree (Figure 3a) as the likely inference due to its minimum number of steps. This hypothesis suggests that TLR 13 is orthologous in archosaurs, likely having arisen through mutation. 

**Figure 4:**
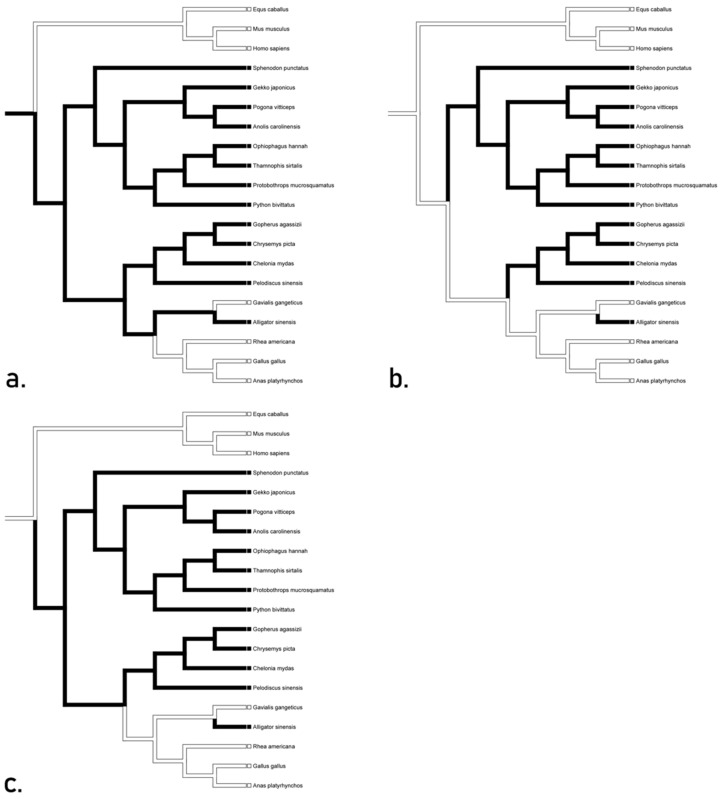
Character optimization of TLR 14 using accelerated (**a**) delayed (**b**), and mixed (**c**) transformations that unequivocally presented the conservation of the TLR 14 throughout Reptilia, along with a loss in *Gavialis*. Accelerated and mixed transformations suggest conservation throughout Reptilia, while delayed transformation hypothesized convergence between major groups.

Character transformations for TLR 14 unequivocally presented the conservation of the gene throughout Reptilia, along with a loss in *Gavialis* (Figure 4). Accelerated and mixed transformations suggest conservation throughout Reptilia, while delayed transformation hypothesized convergence between major groups (i.e., squamates, testudines, and crocodilians; Figure 4b). It is likely that TLR 14 gene may have been present in the basal diapsid. 

**Figure 5:**
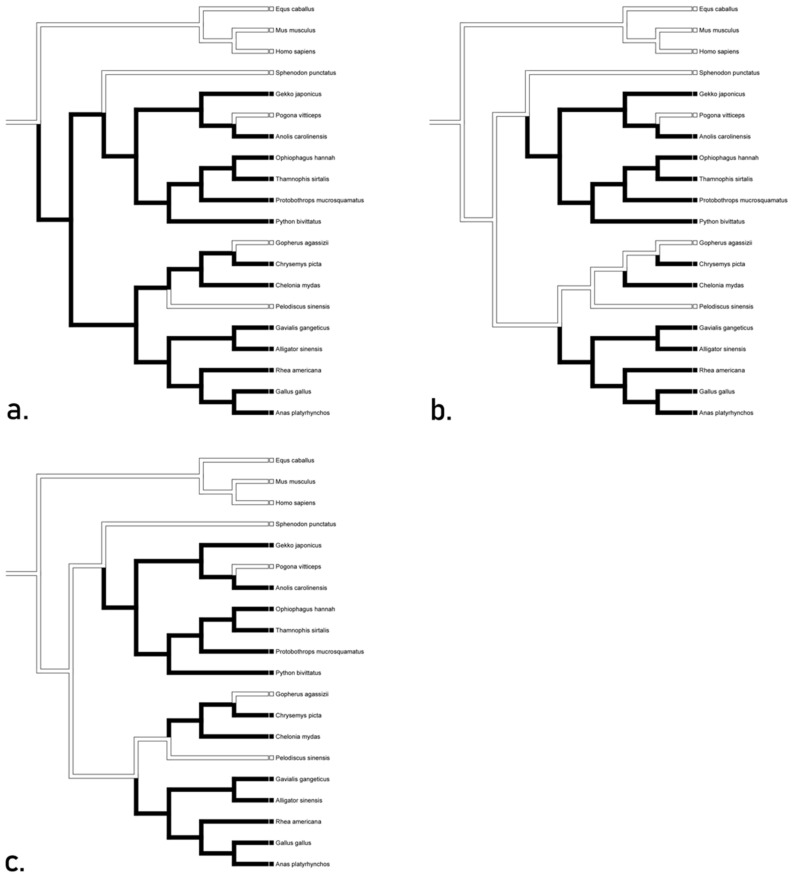
Character optimization of TLR 15 using accelerated (**a**) delayed (**b**), and mixed (**c**) transformations showing its unique evolution in reptilian and avian lineages. Accelerated transformations hypothesize conservation throughout Reptilia with losses in *P. viticeps, G. agassizii, and P. sinesis*, while delayed and mixed inferences suggest convergence between squamates and members of Archosauramorpha as well as a loss in the aforementioned taxa.

**Figure 6:**
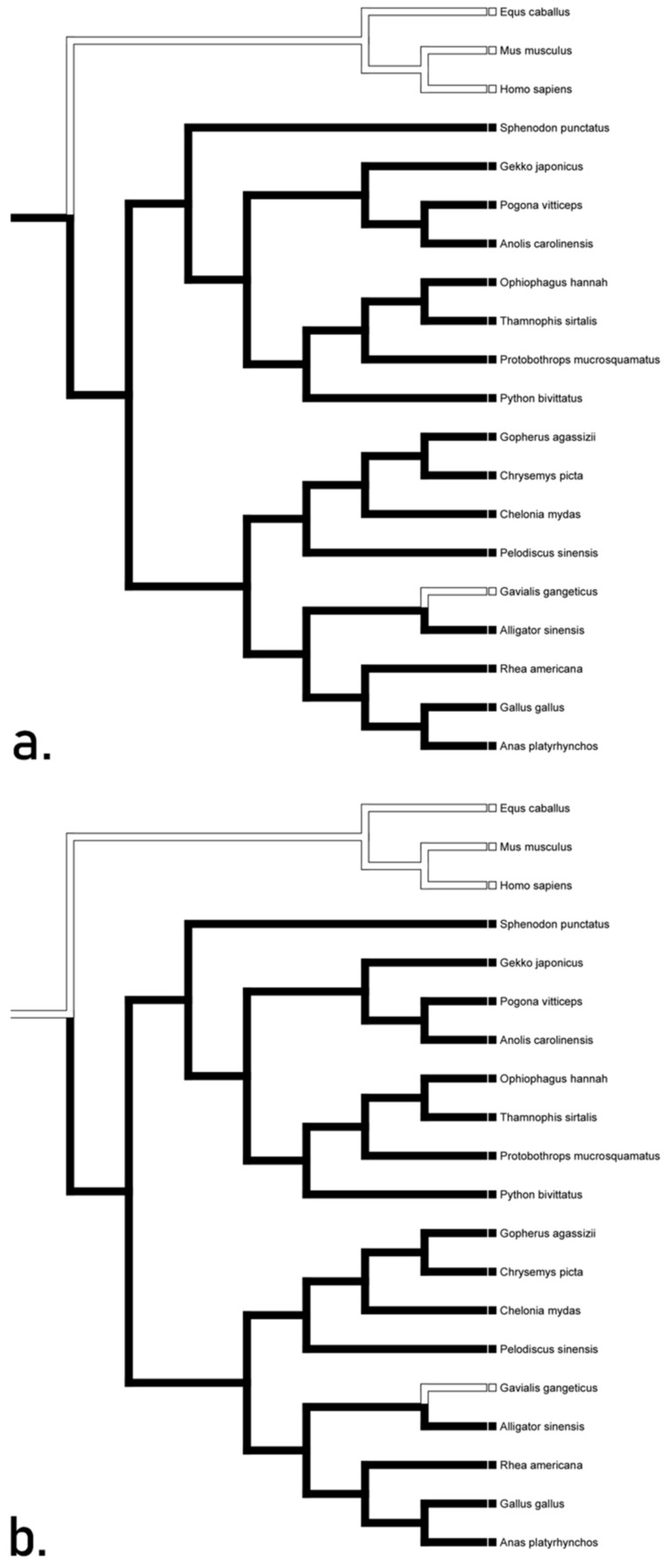
Character optimization of TLR 21 using accelerated transformations (**a**) and delayed (**b**) exhibiting unequivocal loss in *Gavialis* and presence in the basal diapsid.

The evolution of the TLR 15 gene is unique in that it is only recognized in reptilian and avian lineages. Accelerated transformations hypothesize conservation throughout Reptilia with losses in *P. viticeps, G. agassizii, and P. sinesis*, while delayed and mixed inferences suggest convergence between squamates and members of Archosauramorpha as well as a loss in the aforementioned taxa (Figure 5). The sum of these hypotheses suggests that the gene for TLR 15 was not present in the basal diapsid, which corroborates the observation that it is not recognized in any other taxonomic group. The gene for TLR 21 is recognized in all Reptilia; however, it is unequivocally lost in *Gavialis* and present in the basal diapsid (Figure 6).

**Figure 7:**
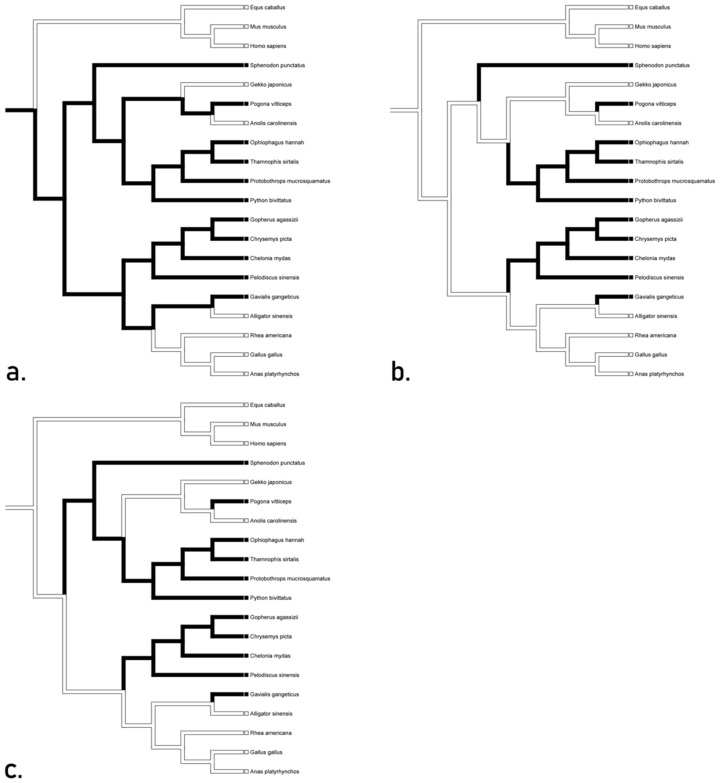
Character optimization of TLR 22 using accelerated (**a**) delayed (**b**), and mixed (**c**) transformations. The accelerated transformation proposed conservation throughout Reptilia with a loss in *Gekko*, *Anolis*, and *Alligator* while delayed transformation also suggests convergence between *Sphenodon*, *Pogona*, *Serpentes*, *Testudines*, and *Gavialis*. Mixed transformation suggests conservation in all Lepidosauria except for losses in *Gekko* and *Pogona*, as well as convergence with and within Archosauria.

The optimization of TLR 22 produced three trees (Figure 7). The accelerated transformation proposed conservation throughout Reptilia with a loss in *Gekko*, *Anolis*, and *Alligator* (Figure 7a). Delayed transformation (Figure 7b) presents the same losses however, it also suggests convergence between *Sphenodon*, *Pogona*, *Serpentes*, *Testudines*, and *Gavialis*. Mixed transformation (Figure 7c) suggests conservation in all Lepidosauria except for losses in *Gekko* and *Pogona*, as well as convergence with and within Archosauria. The overall consensus for these trees suggests that the TLR 22 may have been present in the basal diapsid. 

## 4. Discussion

Alongside contemporary members of Diapsida (Lepidosauramorpha and Archosauramorpha), the diapsid condition (two temporal fenestra) has been displayed by many other diverse groups such as Ichthyosauromorpha, Thalattosauriformes, and Sauropterygia. Among these extinct animals, the earliest presence of the diapsid condition has been identified in members of Araeoscelidia (e.g., *Petrolacosaurus* and *Araeoscelis*) and Neodiapsida (*Youngina capensis*), which includes all modern day avian and non-avian reptiles along with various extinct taxa [19,20]. These stem diapsids occupied a diverse array of ecosystems including both marine and terrestrial habitats, and as such, they may have encountered a diverse array of pathogens, requiring a broad range of functional receptors. Our optimizations suggest that the basal diapsid may have expressed TLRs 1–5, 7, 8, 9, 14, 21, and 22; however, it may not have expressed TLRs 13 and 15. 

The exploration of TLR 13 function has revealed its function to include recognition of a conserved nucleotide sequence of 23S rRNA that is present throughout a variety of both gram-negative and gram-positive bacteria [21]. The gene for TLR 13 is widely distributed throughout vertebrate taxa and is not unique to any specific taxonomic group; however, it is lost in avian lineages and a majority of Archosauramorpha [11]. Our analysis did not support the presence of TLR 13 in the basal diapsid, likely due to the lack of TLR 13 throughout Archosauramorpha. It may be possible that the TLR 13 gene became obsolete in some basal archosaur due to diversification of TLR genes, and the loss of the gene was conserved. Khan et al. [22] found support for significant selective pressures on the evolution of TLR genes in avian lineages because of the host-pathogen interactions experienced by birds. Birds are well known for carrying a wide variety of pathogens, and a basal diapsid may have experienced a similar environment where it dealt with continual exposure to many pathogens. The pressures of diverse pathogen exposure would have led to rapid evolution of TLR genes, potentially making TLR 13 obsolete among other genes. 

Unlike TLR 13, TLR 15 has been found to be unique to reptilian and avian lineages and may have arisen 310 Ma, after the divergence of diapsids and synapsids [10,23]. However, Voogdt et al. [24] found support for the first appearance of TLR 15 as far back as before the divergence of Chondrichthyes and tetrapods, with multiple loss events throughout vertebrate evolution. Current understanding of the function of TLR 15 is that it is responsible for antiviral immune responses, as well as response to bacterial infections [25], with NF-*k*B activation by the binding of proteases [24]. Boyd et al. [23] found that there was significant divergence in the extracellular domains of TLR 15 when compared to that of TLR 1/2, closely related TLR subfamilies, suggesting selective evolution of the receptor. Loss of the TLR 15 receptor in synapsids and retention in the basal diapsid may suggest that the basal diapsid encountered unique pathogens relative to synapsids. It is also possible that the function of TLR 15 is executed by other receptors present in synapsids, such as TLR 1 and TLR 2 [24]. 

The ability of TLRs to recognize a variety of different molecular patterns is well understood. Since the discovery of Toll-protein in *Drosophila*, there have been significant efforts in the field of immunology to characterize interactions between receptors and their respective ligands. As a result, various specific relationships have been revealed between receptors and ligands, such as the aforementioned relationships of the ligands of TLR 13 and TLR 15. However, some ligand recognition is not unique and functional redundancy does occur. For example, a suite of TLRs (TLR 2, TLR 4, TLR 5) have been found to recognize the same bacteria in mice [26]. The redundancy of function in some of these receptors may explain the observed losses of TLRs across Reptilia. Prior analyses of TLR evolution had hypothesized that the maintenance of these receptors is due to selective pressures for function [11]. However, if there is redundancy in function this may facilitate losses as it may conserve energy and resources to lose repetitive receptors. Our analyses also present various cases of reappearances or convergences of these genes throughout the hypothesized trees. Rather than arising independently again, these TLRs are likely convergent due to the homologous nature of these receptors, with losses and reappearances being due to the introduction of minor mutations. 

## 5. Conclusions

Toll-like receptors are among the first responders to pathogens, and as such, the suite of receptors presented by an individual can illuminate certain host-pathogen interactions and constraints. Investigation into the evolution of TLR genes throughout vertebrate lineages can provide a greater understanding of immunological evolution and how environmental constraints may affect the development of immunological traits. Additionally, further exploration into the presence and function of TLR genes throughout reptilian taxa is necessary in order to increase the understanding of immune system function in species threatened by emergent diseases. 

## Data Availability

Data will be uploaded to DRYAD upon acceptance of the manuscript.
